# Geomagnetic and Geoelectric Prospection on a Roman Iron Production Facility in Hüttenberg, Austria *(Ferrum Noricum)*

**DOI:** 10.1002/arp.412

**Published:** 2011-05-12

**Authors:** Georg Walach, Robert Scholger, Brigitte Cech

**Affiliations:** 1 Department Applied Geological Sciences and GeophysicsMontanuniversitaet Leoben, Peter-Tunner-Strasse 25, A-8700 Leoben, Austria; 2 Quaringasse 22/3/7, A-1100 Wien, Austria

**Keywords:** Archaeogeophysics, *Ferrum Noricum*, geomagnetic, geoelectric, archaeology

## Abstract

Geophysical prospection has been applied in the Hüttenberg area (Carinthia, Austria), where important parts of the Roman iron production in the province of Noricum between the first century bc and the fourth century ad are located. A combination of geomagnetic, geoelectric and electromagnetic measurements at different scales yielded information about the extent of the industrial complex and the location of yet undiscovered subsurface monuments in the surrounding area of the Semlach-Eisner archaeological site. The vertical and lateral extension of a slag deposit from the smelting activities could be determined by means of geomagnetic mapping and multi-electrode geoelectric profiles. For the prediction of the continuation of walls in the subsurface outside the excavated area, the total horizontal derivative of the magnetic anomaly as well as geoelectric measurements were most suitable, whereas electromagnetic measurements were not successful owing to the high conductivity of widely spread pieces of slag. Copyright © 2011 John Wiley & Sons, Ltd.

## Introduction

**Figure 1:**
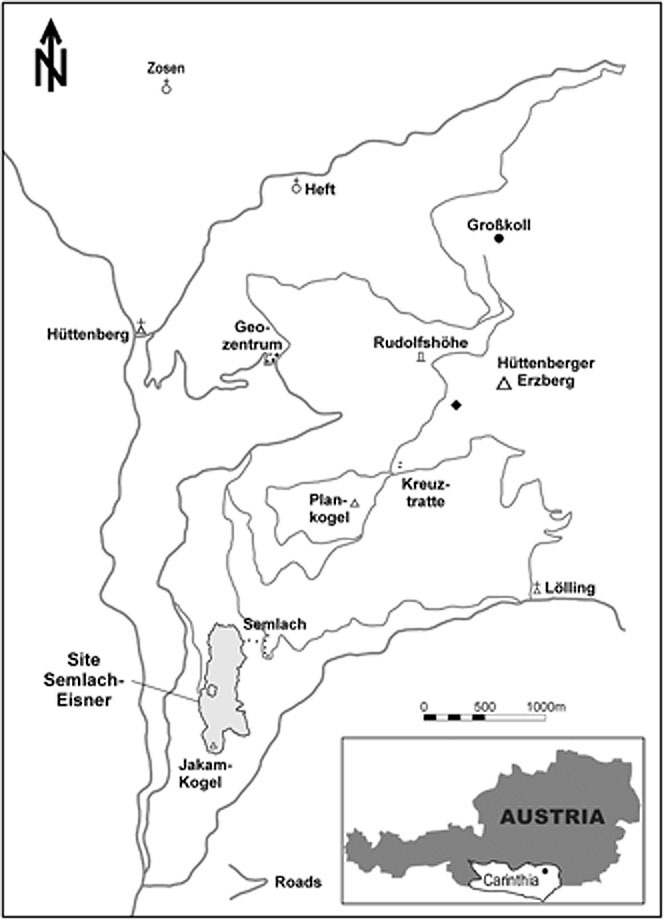
The investigation area of Hüttenberg, Carinthia.

The project is focused on *Ferrum Noricum*, the famous Noric steel, mentioned in numerous Latin and Greek sources from the end of the first century bc, when *Noricum* became a Roman province. The ore deposit of Hüttenberg is famous for its high quality manganiferous ores, which have been mined (according to the current state of research) from the Late Iron Age until 1978, when the last mines were closed. Since the late nineteenth century Roman iron smelting furnaces have been uncovered in the surrounding area and it has been suspected that the main production of Noric steel was located in this area (Figure 1).

Archaeological investigations were focused at the iron production site Semlach-Eisner at an altitude of 962 m on a gently sloping field behind a farmhouse (Figure 1). The archaeological features uncovered so far show that the site comprises an industrial centre for iron production, together with the necessary infrastructure, and dates at least from the end of the first century bc to the middle of the fourth century ad. In the course of the centuries the spatial organization of the site has changed a couple of times (Cech, 2008).

**Figure 2:**
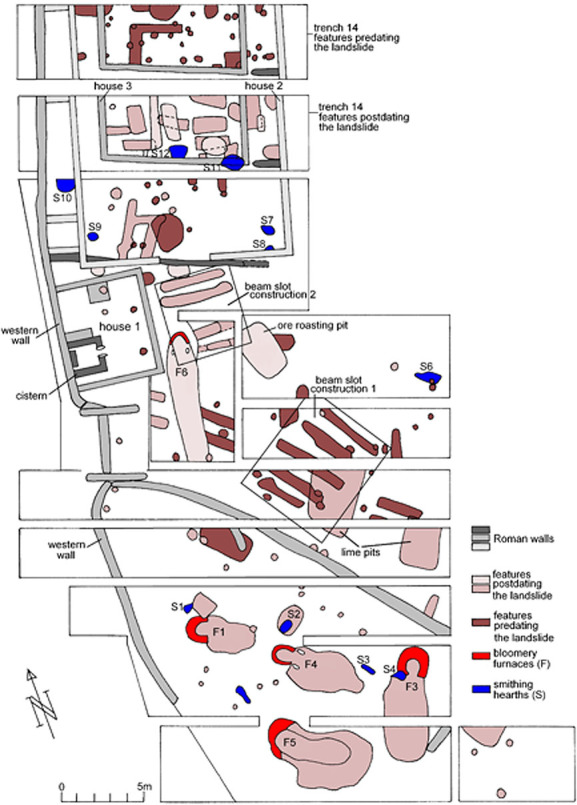
Excavation plan of the Semlach-Eisner site (2010). This figure is available in colour online at http://wileyonlinelibrary.com/journal/arp

Furnaces were abandoned and new ones built; houses with masonry foundations and waddle and daub walls replaced wooden structures; thus the earliest phase consists of beam-slot constructions, postholes and pits sunk into the subsoil. Extensive deforestation in the course of mining and smelting activities led to a landslide around the middle of the first century ad. Evidence of the first anthropogenic activities following the landslide are beam-slot constructions, postholes and pits sunk into the material of the landslide. In the second century ad the wooden constructions were replaced by houses with masonry foundations. The earliest structure identified so far is a cistern, where water from a spring in the hills to the north of the site was collected. When this cistern went out of use, the ground was levelled and a house (house 1) was built on the levelled surface (Figure 2). This house has a mortared floor and a stove for cooking. The finds indicate that this house was used for cooking and administrative purposes. House 3, which was excavated in 2010, belongs to the same chronological phase. The storage hall (house 2) belongs to the last phase of the occupation of this site. Remains of the metallurgical activity excavated so far comprise six furnaces for smelting iron, 12 small smithing hearths for bloom-smithing and an ore roasting pit (Figure 2).

The buildings and pottery finds (local and imported) of glass and other household items, as well as animal bones, provide valuable information about the working and living conditions on a Roman industrial complex.

## Geophysical prospection

The delimitation of monument records in soil by means of geophysical prospection is based on contrasts in petrophysical parameters of the soil compared with the natural soil or underlying bedrock (Aitken, 1974; Scollar *et al*, 1990; Clark, 2001). Geophysical prospection in the Hüttenberg area started in 1990, with geomagnetic and geoelectric surveys (Cech *et al*, 2005; Walach, 2007, 2008). Owing to the variable petrophysical properties of the archaeological objects in the study area, a combination of geomagnetic, geoelectric and electromagnetic measurements was chosen and interpreted relied on an integrated approach (Weymouth, 1985, 1986; Wynn, 1986; Sampson *et al*, 1996; Batayneh *et al*, 2001; Vafidis *et al*, 2005).

- *Geomagnetic measurements*. Remains of the iron production process such as smelting furnaces, smithing hearths and slag heaps, and also stone constructions (Smekalova *et al*, 2005a, 2005b), can be delimited from the natural background by a considerable contrast in their magnetic susceptibility (Crew *et al*, 2002). These variations cause anomalies in Earth's magnetic field, which can be mapped using portable magnetometers (Scollar *et al*, 1986). Earlier geomagnetic measurements on the Semlach-Eisner site revealed that smelting furnaces caused variations of approximately ±250 nT, while anomalies of slag deposits produced values in excess of 2000 nT (Walach, 2008). Accompanying susceptibility measurements of the soil, slag and blocks of the masonry foundations have been undertaken, as well as measurements of the remanent magnetization of excavated burnt soil and rock using oriented samples and standard palaeomagnetic techniques.
- *Geoelectric measurements*. Stone constructions as well as slag deposits of the iron production process are characterized by a relatively high electrical resistivity compared with the underlying soils and rocks (Herbich *et al*, 1997; Dogan and Papamarinopoulos, 2006). Earlier electrical resistance tomography (ERT) surveys conducted on the Semlach-Eisner and Kreuztratte sites (Figure 1) yielded resistivity values for the slag deposits that were two to three times higher than the surrounding soil (Walach, 2007). For the delimitation of the masonry foundations at the Semlach-Eisner site both pole–dipole profiles and ERT have been applied.
- *Electromagnetic measurements*. It can be assumed that the solid rocks of stone constructions have a lower conductivity than the environment (Scollar, 1962; Tabbagh, 1986). For the prospection of building foundations in the excavation area, Geonics EM38 measurements with a penetration depth of 0.75 m (vertical dipole) and 1.5 m (horizontal dipole) have been performed. Geonics EM31 measurements with a penetration depth of about 3 m were also tested.

Three different applications of geophysical prospection are presented: the delimitation of the historical industrial area by means of a geomagnetic ‘walkmag’ survey; the lateral and vertical delimitation of a slag deposit combining geomagnetic and geoelectric data; and the geomagnetic prediction of masonry foundations of a Roman storage hall in the proposed excavation area.

## The delimitation of the Roman iron production facility at Semlach-Eisner

The first evidence for the existence of an iron production facility at the Semlach-Eisner site was suggested by slag finds in a hollow west of the current excavation area. Six furnaces for smelting iron and several associated slag deposits have been found. Further slag finds south of the site provided evidence for a much larger extension of the area of industrialization.

**Figure 3:**
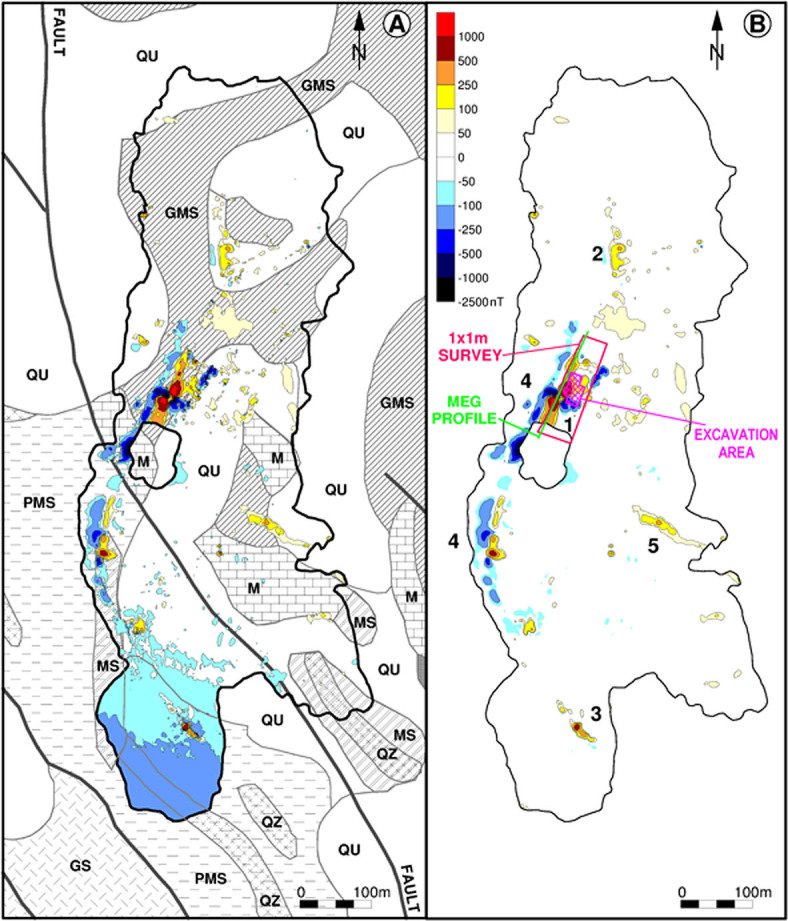
(A) Reduced magnetic anomaly map of the Semiach-Eisner site (geomagnetic ‘walkmag’ survey) with geology (GS, greenschist; PMS, phyllitic mica schist; MS, mica schist; QZ, quartzite; GMS, garnet mica schist; M, marble; QU, Quaternary sediments). (Redrawn after Thiedig *et al*. 1999.) (B) Residual magnetic anomaly map of the Semiach-Eisner site after reductions, trend correction and filtering. This figure is available in colour online at http://wileyonlinelibrary.com/journal/arp

The prospection of large areas requires an efficient and fast method of data acquisition in the field. Therefore, a geomagnetic ‘walkmag’ survey has been undertaken (Smekalova *et al*, 2005a, 2005b) using a GEM 19OH proton magnetometer and a hand-held GPS system; 63,700 data points of the total magnetic intensity (TMI) were collected, covering an area of 400 × 1000 m (Figure 3).

Data processing included reduction of the diurnal variation with reference to a base station located in the survey area (TMI measurements of Earth's magnetic field were taken every 5 min with a Geometrics G856 proton magnetometer), elimination of low quality data according to the instrumental quality index and data affected by technical disturbances (e.g. buildings, fences, etc.).

Geologically, the area is characterized by meta-morphic Koriden rocks of the Eastern Alpine Crystalline series (Clar and Meixner, 1953). Rock types are garnet–mica schist, mica schist and phyllitic mica schist with quartzite, greenschist and marble with a partial superficial cover of Quaternary sediments (Figure 3). In the southernmost part of the survey area, a strong trend in the magnetic data is associated with a geological contact between the Middle and the Upper Austro Alpine units (Gurktal Nappe). Trend reduction was performed by means of calculation of regression surfaces. The residuals of this calculation were filtered with a low-pass filter (Gaussian) and then used for the final presentation of the data. The reduced magnetic anomaly map shows anomalies up to +1000 nT in the excavation area (‘1’ in Figure 3) and indications for further archaeological structures in the measurement field (‘2’ to ‘4’ in Figure 3).

Slag finds around areas ‘2’ and ‘3’ provide strong evidence for the presence of further remains of iron production. The latest results of the geomagnetic prospection indicated that the northern zone (‘2’) represents an industrial area similar to the previously excavated complex (‘1’), although interpretation of this dataset is still in progress. Anomaly zone (‘3’) in the south is consistent with an earlier geomagnetic prospection in this area (Walach, 2008). The magnetic anomaly of the slag heap at the western border of the survey area (‘4’) can be tracked from the excavation area along the hollow towards the south. Enhanced susceptibility values of rocks exposed in the area of anomaly ‘5’ in the eastern part of the study area indicated that this anomaly is related with mineralized rocks (Lafner and Scholger, 2009; Stückler 2010).

The geomagnetic ‘walkmag’ survey at Semlach-Eisner yielded evidence that the Roman industrial zone extends several hundred metres to the north and south of the recent excavation area.

## The slag deposit west of the excavation area

The working area of the Roman iron production facility is bordered to the west by a large wall (‘western wall’ in Figure 2). Beyond this wall the major body of slag can be tracked in the magnetic anomaly map (anomaly zone ‘4’ in Figure 3). Towards the West follows a hollow way, where the first slag finds were reported.

**Figure 4:**
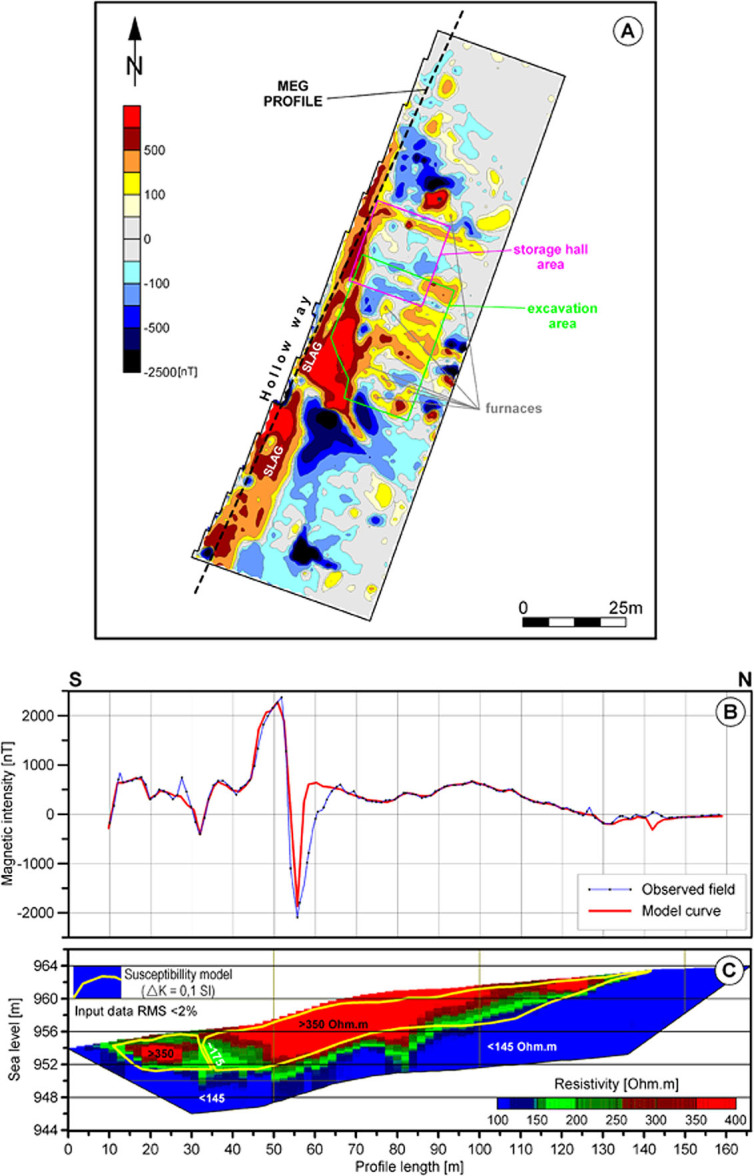
(A) Magnetic anomaly map of the excavation area and the slag zone. Dashed lines indicate the position of geoelectric tomography profiles. (B) Magnetic anomaly (observed field and model curve). (C) Combined resistivity/susceptibility model across the section. This figure is available in colour online at http://wileyonlinelibrary.com/journal/arp

The distribution of the magnetic anomalies based on a 1 × 1m grid magnetic gradiometer survey is shown in Figure 4. The variations inside the slag heap are greater than ±1000 nT, while the furnaces inside the working area yielded maximum anomaly intensity in the range of 300 nT. The magnetic cross-section shown in Figure 4 was calculated from a slice into the 1 × 1 m magnetic grid data at the coordinates of the GEM profile. A large dipole anomaly at metre 55 is caused by a metallic water pipe at shallow depth, which crosses the profile. The susceptibility contrast for the magnetic model is based on *in situ* susceptibility observations of the slag, soil and bedrock. The slag in the Semiach-Eisner area contains an average FeO_*n*_ of 60% (Presslinger, 2008), which accounts for the significant susceptibility difference to the bedrock.

The determination of the depth and volume of the slag deposit was performed by means of multi-electrode geoelectric measurements (MEG) in two parallel profiles with a length of 162 m and an electrode distance of 2 m. Three Wenner configurations (alpha, beta and gamma) were measured, but only Wenner alpha data with an RMS error of less than 2% were used for the inversion (Telford *et at*, 1990; Niesner and Scholger, 2006). The result of the geoelectric inversion indicates a body of about 6 m thickness with high resistivity, which is clearly delimited from the underlying low resistivity bedrock. The interface fits well with the assumptive historical palaeosurface described in the excavation report (Cech, 2008) and with the level of the hollow, which is most likely the base of the slag deposit. The thinning of the body towards both ends of the profile is consistent with the magnetic results. In the southern part of the profile, a smaller slag body is separated by a zone of lower resistivity values, which is possibly related to a later encroachment into the subsurface. The result of the two-dimensional susceptibility modeling (Software Potent by GSS Australia) is in agreement with the resistivity model (Figure 4C). The shape of the magnetic body is quite similar and the small body in the south can also be detached.

Analysis of the volume of the slag by means of geophysical data interpretation may provide evidence for the total quantity of ore smelted. However, due to the complex situation on the Semlach-Eisner site as a result of slag widely dispersed over the investigation area, an estimation of the total volume appears problematic.

## The Roman storage hall on the Semlach-Eisner site

Archaeological excavations on the Semlach-Eisner site yielded remains of a large building, which is assumed to be a Roman storage hall (see house 2 in Figure 2). For the prediction of the further extension of the building to the north, geophysical prospection integrating geomagnetic, geoelectric, and electromagnetic methods was conducted.

**Figure 5:**
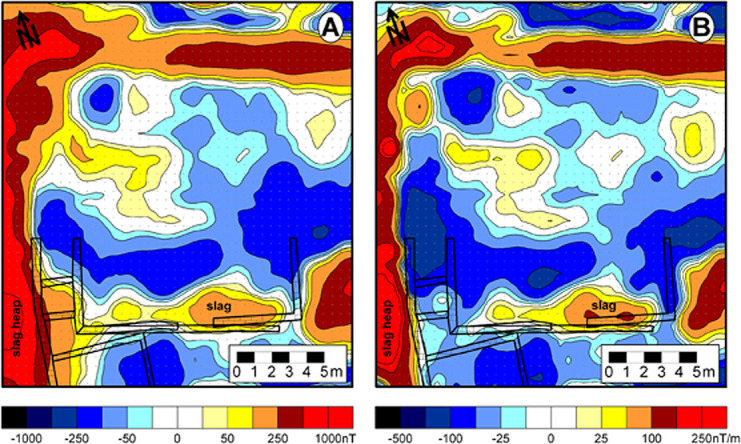
Magnetic anomaly map of the storage hall area and excavated walls. (A) Total magnetic intensity (TMI). (B) Vertical gradient (VG). This figure is available in colour online at http://wileyonlinelibrary.com/journal/arp

Total magnetic intensity (TMI) and its vertical gradient were observed at more than 6100 stations in an investigation area covering 34 × 45 m. Reductions included diurnal variation and elimination of low quality data. As data interpretation should focus on the masonry foundations of house 2, the interpretation area has been reduced to a field of 20 × 22 m covering the suspected walls (see Figure 4 – storage hall area). The reduced magnetic anomalies are presented in Figure 5.

**Figure 6:**
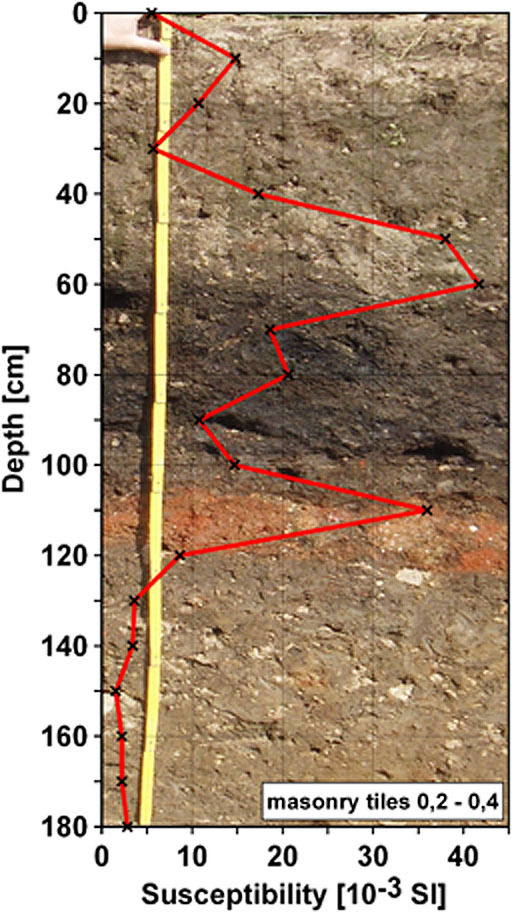
Susceptibility profile from ground surface to base of excavation (trench 14). This figure is available in colour online at http://wileyonlinelibrary.com/journal/arp

Positive values of the anomaly (A) and the vertical gradient (B) represent areas where slag deposits or scattered slag are located (Figure 5). We believe that the negative anomalies are caused by material with lower magnetic susceptibility in the subsurface and, thus, indicate buried walls. This interpretation is based on susceptibility measurements in excavation trench 14 (Figure 2). The foundations of the Roman buildings comprise local metamorphic rocks (e.g. mica schist, marble). Measurements of masonry tiles of houses 2 and 3 yielded an average magnetic susceptibility of 0.2 to 0.4 × 10^−3^ SI, whereas soil and slag typically reach susceptibility values of 2 to 40 × 10^−3^ SI (Figure 6).

In a vertical profile a layered composition of the soil with a wide range of susceptibility values can be observed from the ground surface to the base of the excavation. High susceptibility layers (slag, burned soil) are interbedded with the low susceptibility natural soil.

For the final interpretation of the data, derivative magnetic anomaly maps – reduction to the pole (RTP) and total horizontal derivative (HDR) – have been calculated. Reduction to the pole is a standard technique that recalculates dipolar magnetic anomalies to monopole anomalies over their causative bodies (Telford *et al*, 1990). This technique can simplify the interpretation of the data. The total horizontal derivative represents the slope of a data-surface along lines of fixed direction (profiles). It highlights abrupt changes of the magnetic field. Thus, causative bodies in the subsurface are marked by local minima or maxima of the HDR values over their edges (GETECH, 2007).

**Figure 7:**
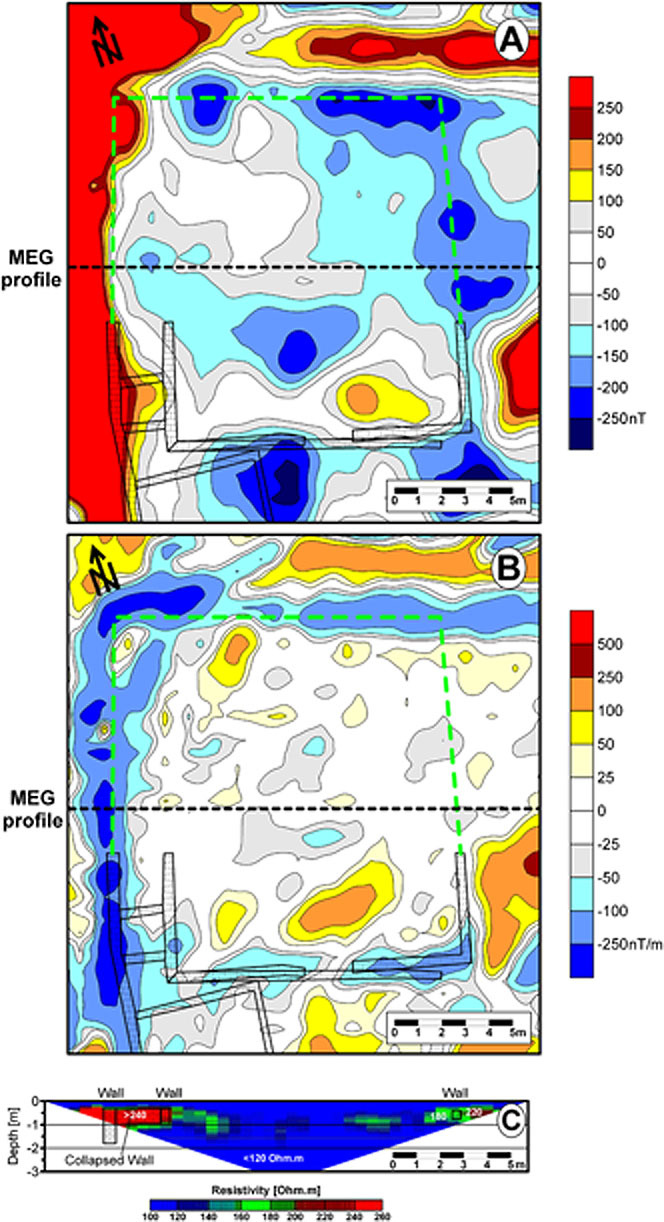
Derivative magnetic anomaly maps of the Roman storage hall on the site Semlach-Eisner. (A) Data reduced to the pole (RTP). (B) Total horizontal derivative (HDR). (C) Resistivity model across the storage hall. This figure is available in colour online at http://wileyonlinelibrary.com/journal/arp

In the RTP image (Figure 7A) negative values (blue to dark blue areas) provide evidence for buried objects (walls) with a lower magnetic susceptibility than the surrounding area; the eastern and northern walls of the storage hall can be identified. Negative anomalies inside the building may be caused by additional walls inside the large storage hall.

The distribution of the HDR (Figure 7B) is calculated from the RTP data. We interpreted negative anomalies of the HDR as evidence for the western, northern and southern walls of the building, while the eastern wall cannot be distinguished from the substrate. Combining both figures, the position of the buried walls can be predicted (green dashed line) fairly well.

Wenner–Beta (dipole–dipole) geoelectric measurements were made in a west–east profile across the storage hall outside the area of excavation, with 42 electrodes and an electrode distance of 0.5 m. The resulting resistivity model (Figure 7C) yielded significant delimitation of the upper and lower borders of the western walls and the collapsed area in between, which is consistent with the excavation results. The eastern end of the profile is characterized by a low contrast between the (small) wall and the surrounding soil. Zones with increased resistivity inside the building are interpreted as internal walls of the depository.

Electromagnetic measurements with the EM38 system were not successful because of the insignificant contrast in conductivity between the walls and the natural environment, and the high conductivity of the widely spread slag.

## Conclusions

The aim of the study was the application of geophysical prospection to different archaeological objects at different scales. Following the prospection concept adopted in the whole study area, a workflow strategy from large-scale to small-scale surveys has been implemented. The delimitation of the industrial complex by means of a geomagnetic ‘walkmag’ survey has shown that structures with an extension of a few metres and an intensity of ±50 nTcan be distinguished, even in a rather noisy environment.

For the location of small-scale archaeological objects (e.g. furnaces) the grid size had to be reduced accordingly. Joint interpretation of geophysical data and excavation results enabled precise determination of the lateral and vertical extent of a slag deposit from the Roman smelting activity. The results fit well with the palaeosurface known from excavation reports and the present topography.

The delimitation of the walls of a large Roman building by means of geomagnetic measurements and interpretation of derivative magnetic maps in combination with ERT profiles proved successful, whereas the electromagnetic method was not suitable because of the high (metallic) conductivity of slag, which is widely spread throughout the investigation area.
